# Dysfunctional immunoproteasomes in autoinflammatory diseases

**DOI:** 10.1186/s41232-016-0011-8

**Published:** 2016-05-28

**Authors:** Hideki Arimochi, Yuki Sasaki, Akiko Kitamura, Koji Yasutomo

**Affiliations:** Department of Immunology and Parasitology, Graduate School of Medicine, Tokushima University, 3-18-15 Kuramoto, Tokushima, 770-8503 Japan

**Keywords:** Autoinflammation, Genetics, Immunoproteasomes

## Abstract

Recent progress in DNA sequencing technology has made it possible to identify specific genetic mutations in familial disorders. For example, autoinflammatory syndromes are caused by mutations in gene coding for immunoproteasomes. These diseases include Japanese autoinflammatory syndrome with lipodystrophy, Nakajo-Nishimura syndrome, joint contractures, muscular atrophy, microcytic anemia, panniculitis-associated lipodystrophy syndrome, and chronic atypical neutrophilic dermatosis with lipodystrophy and elevated temperature syndrome. Causal mutations of these syndromes are present in gene coding for subunits of the immunoproteasome. Importantly, a genetically modified mouse that lacks the catalytic subunit of immunoproteasomes does not always develop an autoinflammatory syndrome. Analysis of causal gene mutations, assessment of patients’ phenotypic changes, and appropriate animal models will be indispensable for clarifying the underlying mechanisms responsible for the development of autoinflammatory syndromes and establishing curative approaches.

## Background

Inflammation is caused by a variety of factors including endogenous abnormalities, infection, and toxins and involved in immune-mediated disorders including autoimmune or autoinflammatory disorders. Autoimmune disorders are caused by hyper-reactivity of the immune system against self-derived antigens [1, 2]. On the other hand, recent studies have uncovered other types of inflammatory pathologies termed “autoinflammation” that are induced by activation of innate immune cells without apparent infection or autoimmune responses. Autoinflammatory syndromes are hereditary diseases and mainly caused by a mutation of a single gene that codes for a component of NACHT, LRR, and PYD domain-containing protein (NLRP) inflammasomes, cytosolic DNA danger sensing machinery, cytokine receptors, and immunoproteasomes [3–5]. However, the causal gene mutations are unclear and/or involve multiple mutations in some cases.

We and the other groups have reported an autoinflammatory syndrome in which the gene for proteasome subunit beta-type 8 (*PSMB8*) has been altered [6–9]. The mutation causes dysfunction of the immunoproteasomes, resulting in the accumulation of ubiquitin-coupled proteins. Although it is now clear that the dysregulation of immunoproteasomes causes an autoinflammatory syndrome, the molecular mechanism of the inflammatory response is unclear. Here, we discuss the recent progress in the genetic analysis of autoinflammatory syndromes caused by dysfunctional immunoproteasomes.

## Main text

### Proteasomes and immunoproteasomes

The proteasome is a multi-subunit protease complex that degrades ubiquitinated proteins in the cytosol for rapid turnover [10, 11]. The 26S standard proteasome is composed of a 19S and 20S proteasome unit. The 20S proteasome is formed by the 14 α subunits and the 14 β subunits, in which β1 (coded by the *PSMB6* gene), β2 (coded by *PSMB7* gene), and β5 (coded by *PSMB5* gene) subunits possess protease activities. The standard proteasome is present in most eukaryotic cells, and the thymoproteasome is a specific proteasome found in the thymus [12]. The immunoproteasome is a special proteasome composed of β1i (coded by *PSMB9*), β2i (coded by *PSMB10*), and β5i (coded by *PSMB8*) subunits instead of the β1, β2, and β5 subunits of the standard proteasome, respectively. The immunoproteasome-specific β subunits are induced by interferon-γ stimulation. The β5i subunit possesses strong chymotrypsin-like activity, and β1i and β2i subunits have caspase and trypsin-like activities, respectively [13]. The immunoproteasome efficiently generates peptides presented by MHC class I and degradation of oxidized proteins to maintain cellular homeostasis [14].

### Autoinflammatory syndromes with proteasome dysfunction

**Fig. 1:**
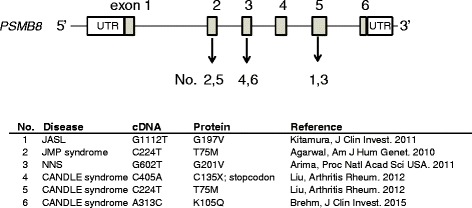
Mutations in *PSMB8*. Causal mutations of the autoinflammatory syndrome in human *PSMB8* gene are listed

Autoinflammatory diseases resulting from dysfunctional proteasomes are termed “proteasome-associated autoinflammatory syndromes” (PRAAS) [15]. PRAAS include Japanese autoinflammatory syndrome with lipodystrophy (JASL) [7], Nakajo-Nishimura syndrome (NNS) [8], joint contractures, muscular atrophy, microcytic anemia, panniculitis-associated lipodystrophy (JMP) syndrome [6], and chronic atypical neutrophilic dermatosis with lipodystrophy and elevated temperature (CANDLE) syndrome [9] (Fig. 1).

JASL is characterized by recurrent fever, nodular erythema, high CRP levels, and hypergammaglobulinemia together with a loss of adipose tissue in the upper part of the body. Three JASL patients from 2 consanguineous families showed high fever and severe inflammation, although they did not possess autoantibodies or an immunocompromised constitution [7]. Patients with the JMP syndrome were found in two family lines in Mexico and Portugal. They showed hepatosplenomegaly; macrosomia; lipodystrophy of face, arms, and chest; sclerodermic skin with erythematous lesions; microcytic anemia with higher serum levels of interferon-γ, IL-8, and IL-6; and hypergammaglobulinemia [6]. NNS is a wasting disease seen early in life and it has been found only in the Japanese population [8]. The patients show elongated and thickened fingers, periodic high fever, nodular erythema, lipomuscular atrophy with joint contractures, myositis, and hypergammaglobulinemia. Cell extracts prepared from the patients indicated that three immunoproteasome-related protease activities were below normal compared with healthy controls. CANDLE syndrome patients have been found in Jewish, Spanish, Caucasian, and Hispanic populations. They also show recurrent fevers; delayed physical development; hypochromic or normocytic anemia; progressive lipodystrophy; joint pain; and increased acute phase reactants with variable clinical features, including skin hyperpigmentation, spot alopecia, and polytrichia [9].

### Causal mutations leading to JASL and other autoinflammatory syndromes with dysfunctional proteasomes

In a search for the causal mutation of JASL, we used SNP homozygosity mapping, linking analysis, and exome analysis [7]. We identified a specific mutation of the *PSMB8* gene present in chromosome 6 in patients from 2 distinct Japanese families. This mutation was homozygous with a transversion of guanine to adenine with an amino acid change from glycine to valine at a position of 197 (p.G197V) of the PSMB8 protein. The original amino acid has been conserved across animal species, suggesting an important role of this amino acid in the maintenance of form and function of the protein.

NNS, JMP, and CANDLE syndromes are also caused by mutations in the *PSMB8* gene, although different regions are involved [6, 8, 9]. Agarwal et al. found a homozygous missense mutation of C to T at position 224 (c.C224T) in the *PSMB8* gene, resulting in a p.T75M change in JMP patients [6]. Arima et al. reported that the causal mutation of NNS is a p.G201V mutation in *PSMB8* exon 5 [8] and Liu and co-workers described one patient with the CANDLE syndrome who possessed a homozygous nonsense mutation at position 405 resulting in a C to A change with a protein truncation. Four others were homozygous and two others had a heterozygous missense mutation at c.C224T. In a Caucasian patient, no mutation in the *PSMB8* gene was found [9]. Recently, new mutations related to the CANDLE syndrome were identified in the *PSMA3* gene coding α7, *PSMB4* coding β7, and *PSMB9* coding β1i as well as *PSMB8* and proteasome maturation protein (*POMP*) genes from 8 patients [16]. One patient had two heterozygous *PSMB4* gene mutations that were c.G(-9)A. in the 5′ UTR and a 9-bp in-frame deletion caused p.D212_V214del. Other *PSMB4* mutations were heterozygous variants c.44insG/p.P16Sfs*45 found in a Jamaican patient and a monoallelic nonsense mutation c.C666A/p.Y222X found in an Irish patient. The mutations in *PSMA3* found in two unrelated patients were heterozygous 3-bp in-frame deletions (c.696_698delAAG/p.R233del) and c.T(404+2)G/p.H111Ffs*10 at the splicing site. The mutation in *PSMB9* was a missense substitution (c.G494A) and affected a conserved amino acid residue (p.G165D). A newly identified *PSMB8* mutation was a heterozygous missense mutation of c.A313C/p.K105Q in an Irish patient. A mutation in the *POMP* gene of a Palestinian patient was a heterozygous frameshift mutation, c.344_345insTTTGA/p.E115Dfs*20. Another mutation in the *PSMB8* gene resulted in a p.Q49K change that was found in patients of juvenile rheumatoid arthritis that had some clinical features similar to NNS [17].

### Effects of PSMB8 protein abnormality on cellular physiology and animal disease models

Mutations found in the *PSMB8* gene from autoinflammatory syndrome patients can affect the PSMB8 protein and immunoproteasome functions. The G201V mutation found in NNS patients affects both the proteolytic activity of β5i and the assembly of the immunoproteasome. These conclusions are based on the observation that an accumulation of immature 20S proteasome precursors was found in NNS cells [7]. Immortalized lymphoblasts derived from a JMP patient showed reduced chymotrypsin-like activity without an effect on trypsin-like or caspase-like activity. Brehm et al. described hematopoietic and non-hematopoietic cells derived from CANDLE patients who had different types of mutations, including those leading to abnormal protein expression, protein folding, proteasome assembly, proteasome activity, and strong type 1 IFN expression [16]. Immortalized transformed B cells from a JASL patient showed lower expression of PSMB8 at the mRNA and protein levels, and the activities of caspase-like, trypsin-like, and chymotrypsin-like proteases in cell extracts were decreased compared with healthy control samples [7]. The reduced expression of mature PSMB8 protein was also confirmed in the skin of a JASL patient. Immature proteasomes were increased in JASL cells, indicating that the immunoproteasome assembly was defective in the mutant cells. Consistent with these data, ubiquitinated proteins were increased in cell extracts of transformed cells and the skin of a JASL patient. IL-6 expression in the skin of the patient and cells carrying mutant PSMB8 proteins were increased via p38 MAP kinase signaling [7]. Kasagi et al. also reported serum IL-6 levels in JASL patients were higher than those of healthy controls [18].

PSMB8 knockout mice grow normally and no spontaneous inflammation is observed, unlike patients with dysfunctional proteasome syndromes. Seifert et al. reported that β5i-deficient mice showed symptoms of experimental allergic encephalomyelitis [14]. However, it has been reported that *PSMB8* deletion or treatment with its specific inhibitor suppresses autoreactive immune responses in murine models of arthritis, diabetes [19], experimental colitis [20], Hashimoto’s thyroiditis [21], and lupus-like disease [22]. These suppressive effects may relate to the ability of β5i-deficient or specific inhibitor-treated cells to diminish Th1, Th2, and Th17 differentiation and enhance regulatory T cell differentiation [23]. The production of IL-23 by activated monocytes and IFN-γ and IL-2 by activated T cells is also blocked by treatment of the cells with a selective inhibitor of β5i [24]. Therefore, to determine how missense mutations in *PSMB8* cause inflammatory responses in human, it will be necessary to establish mice that harbor a mutation in the PSMB8 gene.

## Conclusions

Improvements in genotyping efficiency, sequencing technology, and statistical methodology have made it possible for researchers to identify specific gene mutations associated with autoinflammatory syndromes. Some mutations and polymorphisms connected to dysregulated proteasome syndromes have been reported, but the functional consequences of genetic variations are not fully understood. To increase our understanding of the pathophysiology of these diseases, basic and advanced studies with tissues from patients and genetically modified animals will be required to determine how the mutations affect cellular physiology and proteasome function. Analysis of causal gene mutations, the subsequent phenotypic changes in autoinflammatory syndrome patients, and establishment of a proper animal model for these diseases will be indispensable to clarify the mechanisms of autoinflammatory syndrome development and to develop cures for these diseases.
